# Direct imaging of topological edge states at a bilayer graphene domain wall

**DOI:** 10.1038/ncomms11760

**Published:** 2016-06-17

**Authors:** Long-Jing Yin, Hua Jiang, Jia-Bin Qiao, Lin He

**Affiliations:** 1 Center for Advanced Quantum Studies, Department of Physics, Beijing Normal University, Beijing 100875, China; 2 College of Physics, Optoelectronics and Energy, Soochow University, Suzhou 215006, China

## Abstract

The AB–BA domain wall in gapped graphene bilayers is a rare naked structure hosting topological electronic states. Although it has been extensively studied in theory, a direct imaging of its topological edge states is still missing. Here we image the topological edge states at the graphene bilayer domain wall by using scanning tunnelling microscope. The simultaneously obtained atomic-resolution images of the domain wall provide us unprecedented opportunities to measure the spatially varying edge states within it. The one-dimensional conducting channels are observed to be mainly located around the two edges of the domain wall, which is reproduced quite well by our theoretical calculations. Our experiment further demonstrates that the one-dimensional topological states are quite robust even in the presence of high magnetic fields. The result reported here may raise hopes of graphene-based electronics with ultra-low dissipation.

## Abstract (web summary)

Domain wall between gapped graphene bilayers is believed to host one-dimensional topological states, which is yet waiting for direct evidences. Here, Yin *et al.* report images of the AB-BA stacked bilayer graphene domain wall, providing direct evidence for topological edge states in such system.

## Graphical abstract

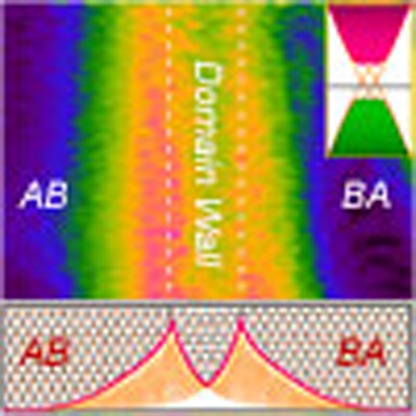

Looking for systems where topological edge states persist in the absence of external magnetic fields boosts rapid developments in condensed matter physics in the past few years^1^,^2^,^3^,^4^,^5^,^6^,^7^,^8^,^9^,^10^,^11^,^12^,^13^,^14^,^15^. Gapped graphene bilayer with smooth domain walls is predicted to be one of the most promising candidates where charge carriers can travel long distances with ultra-low dissipation^8^,^9^,^10^,^11^,^12^. The domain wall separating two oppositely biased bilayer graphene is first proposed by Martin *et al.*^8^ to host one-dimensional (1D) topological states. Later, the domain wall between AB- and BA-stacked bilayer graphene under a uniform external field is demonstrated to be equivalent to the gate-polarity domain wall^8^ and it is believed to be a crystalline topological defect hosting symmetry-protected topological gapless mode because of a change in the Chern number^12^. Very recently, the existence of topologically protected 1D edge states have been demonstrated explicitly in the two types of domain walls through transport measurement^13^,^14^, opening up opportunities for exploring unique topological states in graphene bilayer.

The AB–BA domain wall in graphene bilayer, with electrons residing right at the surface, provides unprecedented opportunities to directly image the topologically protected 1D conducting channels. More importantly, such a crystalline topological line defect exists naturally in Bernal graphene bilayers grown by chemical vapour deposition^16^,^17^ and in exfoliated bilayer graphene (that is, prepared using adhesive tape) from graphite^13^. Here we report direct imaging of the topologically protected 1D conducting channels in the AB–BA domain wall in exfoliated graphene bilayer. The exfoliated bilayer and trilayer graphene flakes were deposited on the substrate (here the supporting substrate is graphite) during the process of mechanical exfoliation and, very importantly, these graphene sheets decouple from the graphite surface due to the presence of the stacking misorientation with the underlying substrates, as demonstrated in this study and in previous studies^18^,^19^,^20^,^21^,^22^,^23^,^24^,^25^.

## Results

### The structure of AB–BA stacking domain wall

**Figure 1:**
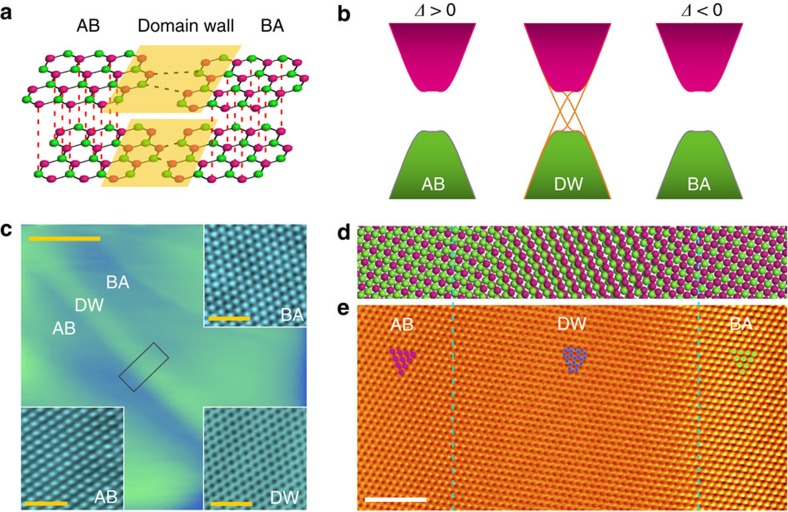
AB–BA domain wall in bilayer graphene. (**a**) Schematic representation of an AB–BA domain wall in bilayer graphene. (**b**) Schematic band structures of the AB, domain wall (DW) and BA regions of a bilayer graphene. The AB- and BA-stacked regions are gapped. The topological edge states (orange curves) emerge in the DW region. (**c**) STM topographic image (80 × 80 nm) of a decoupled bilayer graphene region on graphite surface (*V*_b_=0.4 V, *I*=0.25 nA). An AB–BA domain wall is observed in the bilayer. Scale bar, 20 nm. Insets: atomic-resolution STM images in the AB, DW and BA regions, respectively. Scale bars, 1 nm. (**d**) Schematic image of the AB–BA domain wall. (**e**) A typical atomic-resolution STM current image across the AB–BA domain wall (rectangular region in **c**). A transition from triangular lattice (in the AB region) to hexangular-like lattice (in the centre of the domain wall) and then to triangular lattice (in the BA region) is clearly observed. The width of the domain wall is estimated to be (8±1) nm. Scale bar, 2 nm.

**Figure 2:**
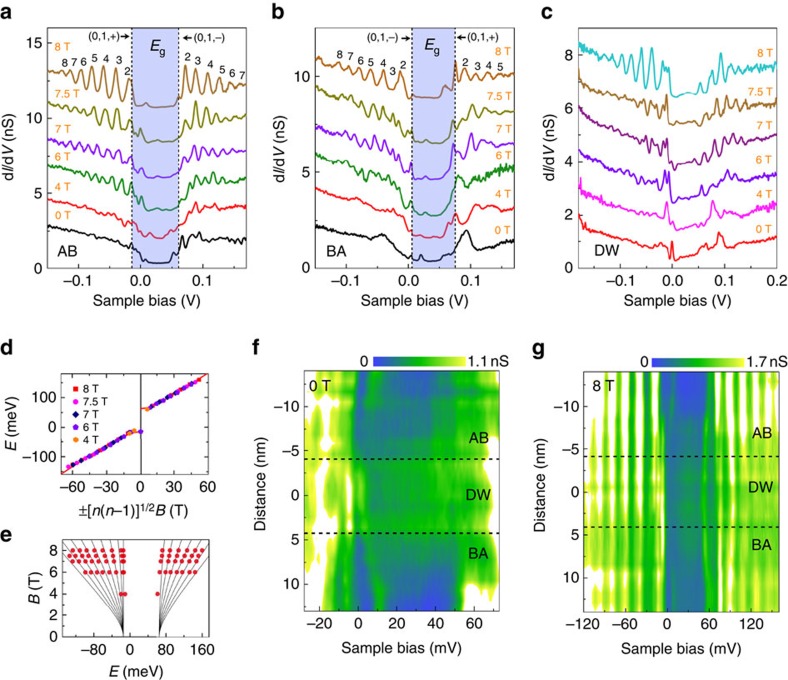
Microscopic properties of the AB–BA domain wall. (**a**–**c**) Tunnelling spectra of the gaped graphene bilayers recorded at the AB-stacked region (**a**), the BA-stacked region (**b**) and the domain wall region (**c**) under various magnetic fields. LL peak indices are marked (± are valley indices) and the gap are labelled by shadows in the AB and BA bilayer regions. The tunnelling curves are offset in *y* axis for clarity. (**d**,**e**) LL peak energies extracted from **a** plotted versus ±[*n*(*n*−1)]^1/2^*B* (**d**) and the magnetic fields *B* (**e**). The solid curves are the fitting of the data with the theoretical equation (equation (1)), yielding the band gap of *E*_g_=80±1 meV and effective mass of m*=(0.0454±0.0001)*m*_*e*_ (*m*_*e*_ is the free-electron mass). (**f**,**g**) STS spectra maps at 0 T (**f**) and 8 T (**g**) measured across the AB–BA domain wall. The zero position is defined at the middle of the domain wall.

To identify the AB–BA domain wall in decoupled bilayer graphene on graphite, we used both the scanning tunnelling microscopy (STM) images (Fig. 1) and the scanning tunnelling spectroscopy (STS) spectra (Fig. 2). First, the decoupled bilayer graphene on graphite exhibits a small period of moiré patterns (that is, with a large rotation angle with the substrate) in the STM measurements^19^,^21^,^25^ and its atomic-resolution STM image shows a triangular lattice because of the A/B atoms' asymmetry in the topmost Bernal bilayer (see Fig. 1c and Supplementary Fig. 1). The decoupled monolayer graphene also exhibits a small period of moiré patterns; however, its atomic-resolution STM images show a hexagonal lattice (see Supplementary Fig. 1). The high-field STS spectra provide further information about the stacking orders of the topmost few layers^22^: the decoupled Bernal bilayer shows Landau quantization of massive Dirac Fermions (Fig. 2 and Supplementary Fig. 2)^22^,^26^, whereas the decoupled monolayer exhibits Landau quantization of massless Dirac fermions (see Supplementary Fig. 1)^18^,^19^.

Once the decoupled bilayer graphene is identified, we used STM measurements to find 1D structures (see Fig. 1c as an example) in the bilayer region as a possible candidate for the AB–BA domain wall. The strong dependence of the 1D structure on the bias voltage (used for imaging), as shown in Supplementary Fig. 3, excludes the graphene nanoripple^27^ or nanowrinkle^28^ as the origin of the 1D structure. We attribute the 1D structure in Fig. 1c to the AB–BA domain wall in bilayer graphene. We can observe the AB–BA domain walls in the STM measurement, owing to their relatively higher conductivity comparing with that of the adjacent gapped bilayer regions (see Fig. 2f). The ultra-low random potential fluctuations due to substrate imperfections allows us to obtain high-quality atomic-resolution STM images of the domain wall, as shown in Fig. 1c,e. We obtained a triangular lattice, as is the characteristic of Bernal-stacked bilayer, both in the left and right regions, whereas we obtained a hexagonal-like lattice in the centre of the domain wall (see the insets of Fig. 1c). The 1D structure with hexagonal-like lattice at its centre, and AB and BA domains surrounding directly demonstrated that the studied structure is the AB–BA domain wall. Figure 1d shows a representative atomic-resolution STM image of the AB–BA domain wall. From left to right of the domain wall, the two graphene sheets translate relative to each other in opposite directions (one translates downward and the other translates upward), completing an interlayer translation from AB to BA stacking. The interatomic distances in the domain wall and in the Bernal bilayer regions were further analysed by taking a two-dimensional Fourier transform and the interatomic distances in the domain wall are (1.5±0.5)% smaller than those in the surrounding Bernal regions (Supplementary Fig. 4). To complete a one-bond-length armchair-direction interlayer translation from AB to BA stacking, the width of the domain wall is estimated to be ∼9.0 nm with the the measured lattice deformation ∼1.5%, which agrees quite well with the measured value ∼(8.0±1.0) nm. The angle between the boundary normal and the translation direction is measured to be about 85°, indicating that the studied domain wall is almost a purely shear soliton^16^.

### Microscopic electronic properties of the AB–BA domain wall

The electronic properties around the AB–BA domain wall are further studied by STS measurements, as shown in Fig. 2 and Supplementary Figs 5 and 6. The spectra recorded in both the AB- and BA-stacked regions exhibit characteristics that are expected to be observed in gapped graphene bilayers^21^,^26^,^29^. The substrate breaks the inverse symmetry of the topmost adjacent bilayers and then a finite gap ∼80 meV is generated in the parabolic bands of the Bernal bilayer (the charge neutrality points of the two Bernal bilayer regions are measured to differ ∼15 meV). At the level of low-energy effective theory, the AB-stacked bilayer is equivalent to the BA-stacked bilayer subjecting to the opposite gate polarity^12^. Thus, the sign of the energy gap changes across the domain wall from the AB- to BA-stacked regions and symmetry-protected gapless modes are expected to emerge in the domain wall. In high magnetic fields, the spectra recorded in the Bernal bilayer regions exhibit Landau quantization of massive Dirac fermions (Fig. 2d,e and see Methods and Supplementary Information for further analysis). The two lowest Landau levels (LLs) LL_(0,1,+)_ and LL_(0,1,−)_ (here 0/1 are Landau indices and +/− are valley indices), which are a couple of layer-polarized quartets, depends on the sign of the gate polarity (or the sign of the energy gap) of the two Bernal bilayer regions^29^. Therefore, they are reversed in the adjacent AB- and BA-stacked regions, as shown in Fig. 2a,b. In the experiment, the measured local density of states (LDOS) at position *r* are determined by the wavefunctions, whereas the wavefunctions of LLs have their spatial extent, ∼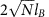 (here *N* is the Landau index and 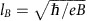 which is of the order of 10 nm for the magnetic fields applied in our experiment)^26^. Consequently, we can detect LLs of both the AB and BA domains in the spectra recorded in the AB–BA domain wall (Fig. 2c and see Supplementary Fig. 5 for details of calculation). The ‘splitting' of the LLs recorded in the domain wall, as shown in Fig. 2c,g, arises from the relative shift of the charge neutrality points of the adjacent AB and BA domains. By using similar STM measurements, layer stacking domain walls in trilayer graphene, which separate ABA- and ABC-stacked trilayer graphene, have also been observed unambiguously in our experiment (see Supplementary Figs 7 and 8 for an example). Our result demonstrates that the layer-stacking domain walls naturally exist in graphene multilayers and affect their electronic properties dramatically.

### Mapping of the topological states at the AB-BA domain wall

**Figure 3:**
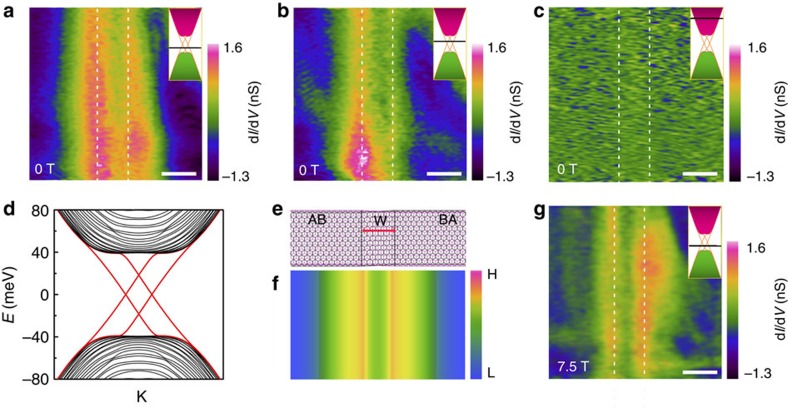
Direct imaging of the 1D conducting channels at the AB–BA domain wall. (**a**–**c**) d*I*/d*V* maps recorded under 0 T along the AB–BA domain wall with the fixed sample bias of 30 mV (**a**), 40 mV (**b**) and 300 mV (**c**), respectively. The 1D topological states are predominantly located at the two edges of the domain wall. (**d**) A representative theoretical band structure of an AB–BA domain wall with width of 8 nm and the gap in the Bernal region of 80 meV. (**e**) Illustration of an AB–BA domain wall. (**f**) Spatial distribution of the topological states around the domain wall obtained by theoretical calculation. (**g**) STS map of the domain wall taken at 7.5 T with sample bias of 30 mV. The two edges of the AB–BA domain wall are labelled by dashed lines. Scale bars, 10 nm.

To further confirm the existence of symmetry-protected topological conducting channels in the AB–BA domain wall, we directly imaged these 1D states by operating energy-fixed STS mapping, which reflects the LDOS in real space. Figure 3a–c shows several STS maps at different energies. At the energies within the band gap of the adjacent AB and BA domains, clearly 1D conducting channels can be observed along the domain wall. A notable feature of the topological states is that they mainly located at the two edges of the AB–BA domain wall and such a feature is independent of the energy of the gapless edge states. To verify the spatial distribution of the gapless states, we calculated electronic structures of a shear domain wall with a finite width *W* (see Fig. 3d for an example). Figure 3e shows a schematic representation of the domain wall. In the calculation, we consider a tight-binding Hamiltonian with nearest-neighbour intra- and interlayer hopping and a finite chemical potential difference between two layers is taking into account to describe the energy gap observed in the Bernal regions^12^. The symmetry-protected gapless edge states emerge in the domain wall (Fig. 3d), which is irrespective of the type and width of the domain wall (see Methods and Supplementary Information for details of calculation). In our experiment, the STS maps probe predominantly the LDOS of the top layer. To compare with the experimental result, we plot a representative theoretical distribution of the gapless edge states in the topmost graphene layer in Fig. 3f. Obviously, the topological states are mainly located at the two edges of the domain wall and this feature is found to be independent of the probed energy within the gap of the Bernal bilayer regions. Here we should point out that such a spatial distribution of the topological states is independent of the edges of the domain wall (see Supplementary Fig. 9). Therefore, our experimental observations are reproduced quite well by the theoretical calculations. This provides direct and compelling evidence that the symmetry-protected topological edge states exist in the AB–BA domain walls of gapped bilayer graphene. In the experiment, the distributions of the edge states reveal notable asymmetry at various setting energies (Fig. 3a,b). This may result from the energy difference of the charge neutrality points between the adjacent AB and BA domains (∼15 meV in our sample) or the existence of the disorder potential near the domain wall (see Supplementary Fig. 10 for details of discussion).

## Discussion

The STS maps of the gapless edge states are also measured in the presence of high magnetic fields (Fig. 3g). It is remarkable that these states are quite robust even in the highest magnetic field ∼8 T of our STM system. More importantly, the full width at half-maximum of topological states along the two edges of the domain wall decreases with increasing the magnetic fields (Supplementary Fig. 11), which may further diminish any possible scattering of the topological edge states along the AB–BA domain walls. In a very recent transport measurement, it was also demonstrated that the topological feature of the gapless edge states is very robust against the perturbation of external magnetic fields and the backscattering of the topological states is further suppressed in the presence of magnetic fields^14^. Our work thus demonstrates the robust feature of the symmetry-protected topological edge states in the AB–BA domain walls of gapped bilayer graphene, opening a wide vista of graphene-based topological transport properties.

## Methods

### Sample preparation and STM/STS measurements

The measurements were employed on highly oriented pyrolytic graphite surface. The highly oriented pyrolytic graphite samples were of ZYA grade (from NT-MDT) and were surface cleaved immediately by the adhesive tape method before experiments without any further processing. The STM system was an ultrahigh vacuum scanning probe microscope (USM-1500S) from UNISOKU with the magnetic fields up to 8 T. All the STM and STS measurements were performed in the ultrahigh vacuum chamber (∼10^−11^ Torr) with constant-current scanning mode. The experiments were acquired at temperature ∼4.5 K. The STM tips were obtained by chemical etching from a wire of Pt(80%) Ir(20%) alloys. Lateral dimensions observed in the STM images were calibrated using a standard graphene lattice and a Si (111)-(7 × 7) lattice and Ag (111) surface. The d*I/*d*V* measurements were taken with a standard lock-in technique by turning off the feedback circuit and using a 793-Hz 5 mV a.c. modulation of the sample voltage. All the STS measurements were not recorded until the right standard tunnelling spectra of graphite was obtained.

### Landau quantization in bilayer graphene

The LL sequences of gapped graphene bilayers can be described by^29^


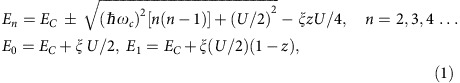


where *E*_C_ is the energy of charge neutrality point, *ω*_*c*_=*eB*/m* is the cyclotron frequency, m* is the effective mass of charge carriers and *ξ*=± are the valley indices. We have *z*=2*ħω*_*c*_/*t*_⊥_<<1 for *B*≤8 T and |*U*|≈*E*_g_ (gap energy) when the interlayer bias *U*<*t*_⊥_. The fitting results of experimental data to equation (1) are shown in Fig. 2 and Supplementary Fig. 2.

### Calculation of the topological edge states in the AB–BA domain walls

To better understand the phenomena observed in the experiment, we further perform the numerical studies of the kink states in the AB–BA domain wall for comparison. We consider two typical AB–BA sandwich structures: (i) zigzag domain wall as illustrated in Supplementary Fig. 9d and (ii) armchair domain wall as illustrated in Supplementary Fig. 9a^30^. The domain wall in any geometry can be regarded as the mixture of these two typical domain wall structures. For both two structures, the domain wall region locates in 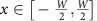. The tight-binding Hamiltonian for AB-stacked bilayer Graphene region can be written as^12^:


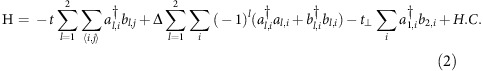


where 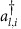
(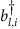) is the creation operator for electron on site *i*=(*x*,*y*) in layer *l* on sublattice *A* (*B*); *l*=1 and 2 denote the top and bottom layer, respectively; *t* and *t*_⊥_ are the intralayer and interlayer hopping energy between nearest-neighbour sites; and Δ is the chemical potential between the top and bottom layer. These parameters are set as *t*=2.8 eV, *t*_⊥_=0.4 eV and Δ=0.06 eV. For BA-stacked region, the model is similar as equation (2), except that the interlayer term is replaced by 
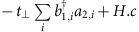
. For the domain wall region, owing to the lattice misalignment, the interlayer hopping energy is much weaker than that in the AB(BA)-stacked region. We also describe such region by equation (2) but set *t*_⊥_=0. In the following numerical calculations, for the sake of the simplicity, both AB- and BA-stacked regions are modelled by the wide nanoribbon with open boundary condition. For the zigzag structure (see Supplementary Fig. 9), the whole system width is 264 nm and the domain wall width is *W*=8.52 nm. For the armchair structure (see Supplementary Fig. 9), the whole system width is 156 nm and the domain wall width is *W*=8.40 nm. First, we obtain the band structure for the domain wall structure. The studied system has the translational symmetry along the *y* direction and the momentum *k* in the *y* direction is a good quantum number. Performing the partial Fourier transformation in *y* direction, one obtain the Hamiltonian *H*(*k*,*l*,*x*) under the bases of sites [*l*,*x*]. Here, *l* denotes the layer index and *x* denotes the position in *x* direction. The energy band can be obtained by directly diagonalizing the *H*(*k*,*l*,*x*). Next we solve the eigenmode of the system at fixed energy *E*. If *E* collide with energy band at *k*_*a*_=*k*_*1*_, *k*_*2*_, …*k*_*M*_, the system has *M* eigenmode. The eigenstate of mode *k*_a_ can be obtained by solving the eigen equation *H*(*k*_a_,*l*,*x*)Ψ_l,a_(*x*)=*E*Ψ_l,a_(*x*). The spatial distribution of the *k*_a_ mode is characterized by 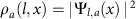. If *ρ*_*a*_(*l*, *x*) mainly locates around the domain wall, *k*_a_ mode is the kink state. If *ρ*_a_(*l*, *x*) is nearly uniform distributed in the whole sample, *k*_a_ mode is the bulk state. Finally, the spatial distribution for the carrier density of states in top layer at energy *E* is calculated from 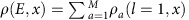. In principle, *ρ*(*E*, *x*) should be directly proportional to STM signal d*I*/d*V* at position *x* and energy *E*, which had been measured in our experiments.

The data that support the findings of this study are available from the corresponding author on request.

## Additional information

**How to cite this article:** Yin, L.-J. *et al.* Direct imaging of topological edge states at a bilayer graphene domain wall. *Nat. Commun.* 7:11760 doi: 10.1038/ncomms11760 (2016).

## Supplementary Material

Supplementary Information Supplementary Figures 1-11

## References

[b1] Kane C. L. & Mele E. J. Quantum spin Hall effect in graphene. Phys. Rev. Lett. 95, 226801 (2005). 16384250 10.1103/PhysRevLett.95.226801

[b2] Kane C. L. & Mele E. J. Z_2_ topological order and the quantum spin Hall effect. Phys. Rev. Lett. 95, 146802 (2005). 16241681 10.1103/PhysRevLett.95.146802

[b3] Bernevig B. A., Hughes T. L. & Zhang S.-C. Quantum spin Hall effect and topological phase transition in HgTe quantum wells. Science 314, 1757–1761 (2006). 17170299 10.1126/science.1133734

[b4] König M. *et al.* Quantum spin Hall insulator state in HgTe quantum wells. Science 318, 766–770 (2007). 17885096 10.1126/science.1148047

[b5] Yu R. *et al.* Quantized anomalous Hall effect in magnetic topological insulators. Science 329, 61–64 (2010). 20522741 10.1126/science.1187485

[b6] Chang C.-Z. *et al.* Experimental observation of the quantum anomalous Hall effect in a magnetic topological insulator. Science 340, 167–170 (2013). 23493424 10.1126/science.1234414

[b7] Du L., Knez I., Sullivan G. & Du R. R. Robust helical edge transport in gated InAs/GaSb bilayers. Phys. Rev. Lett. 114, 096802 (2015). 25793839 10.1103/PhysRevLett.114.096802

[b8] Martin I., Blanter Y. & Morpurgo A. Topological confinement in bilayer graphene. Phys. Rev. Lett. 100, 036804 (2008). 18233021 10.1103/PhysRevLett.100.036804

[b9] Jung J., Zhang F., Qiao Z. & MacDonald A. H. Valley-Hall kink and edge states in multilayer graphene. Phys. Rev. B 84, 075418 (2011).

[b10] Zhang F., MacDonald A. H. & Mele E. J. Valley Chern numbers and boundary modes in gapped bilayer graphene. Proc. Natl Acad. Sci. USA 110, 10546–10551 (2013). 23754439 10.1073/pnas.1308853110 PMC3696819

[b11] Li X., Zhang F., Niu Q. & MacDonald A. H. Spontaneous layer-pseudospin domain walls in bilayer graphene. Phys. Rev. Lett. 113, 116803 (2014). 25259998 10.1103/PhysRevLett.113.116803

[b12] Vaezi A., Liang Y., Ngai D. H., Yang L. & Kim E.-A. Topological edge states at a tilt boundary in gated multilayer graphene. Phys. Rev. X 3, 021018 (2013).

[b13] Ju L. *et al.* Topological valley transport at bilayer graphene domain walls. Nature 520, 650–655 (2015). 25901686 10.1038/nature14364

[b14] Li J. *et al.* Experimental observation of edge states at the line junction of two oppositely biased bilayer graphene. Preprint at arXiv:1509.03912 (2015).

[b15] Qiao Z. *et al.* Current partition at topological channel intersections. Phys. Rev. Lett. 112, 206601 (2014).

[b16] Alden J. S. *et al.* Strain solitons and topological defects in bilayer graphene. Proc. Natl Acad. Sci. USA 110, 11256–11260 (2013). 23798395 10.1073/pnas.1309394110 PMC3710814

[b17] Butz B. *et al.* Dislocations in bilayer graphene. Nature 505, 533–537 (2014). 24352231 10.1038/nature12780

[b18] Li G., Luican A. & Andrei E. Y. Scanning tunneling spectroscopy of graphene on graphite. Phys. Rev. Lett. 102, 176804 (2009). 19518809 10.1103/PhysRevLett.102.176804

[b19] Luican A. *et al.* Single-layer behavior and its breakdown in twisted graphene layers. Phys. Rev. Lett. 106, 126802 (2011). 21517338 10.1103/PhysRevLett.106.126802

[b20] Song Y. J. *et al.* High-resolution tunnelling spectroscopy of a graphene quartet. Nature 467, 185–189 (2010). 20829790 10.1038/nature09330

[b21] Miller D. L. *et al.* Observing the quantization of zero mass carriers in graphene. Science 324, 924–927 (2009). 19443780 10.1126/science.1171810

[b22] Yin L.-J., Li S.-Y., Qiao J. -B., Nie J.-C. & He L. Landau quantization in graphene monolayer, Bernal bilayer, and Bernal trilayer on graphite surface. Phys. Rev. B 91, 115405 (2015).

[b23] Xu R. *et al.* Direct probing of the stacking order and electronic spectrum of rhombohedral trilayer graphene with scanning tunnelingmicroscopy. Phys. Rev. B 91, 035410 (2015).

[b24] Yin L.-J., Qiao J.-B., Zuo W.-J., Li W.-T. & He L. Experimental evidence for non-Abelian gauge potentials in twisted graphene bilayers. Phys. Rev. B 92, 081406 (R) (2015).

[b25] Yin L.-J. *et al.* Landau quantization and Fermi velocity renormalization in twisted graphene bilayers. Phys. Rev. B 92, 201408 (R) (2015).

[b26] Yin L.-J., Zhang Y., Qiao J.-B., Li S.-Y. & He L. Experimental observation of surface states and Landau levels bending in bilayer graphene. Phys. Rev. B 93, 125422 (2016).

[b27] Bai K.-K. *et al.* Creating one-dimensional nanoscale periodic ripples in a continuous mosaic graphene monolayer. Phys. Rev. Lett. 113, 086102 (2014). 25192109 10.1103/PhysRevLett.113.086102

[b28] Yan W. *et al.* Strain and curvature induced evolution of electronic band structures in twisted graphene bilayer. Nat. Commun. 4, 2159 (2013). 23851673 10.1038/ncomms3159

[b29] Rutter G. M. *et al.* Microscopic polarization in bilayer graphene. Nat. Phys. 7, 649–655 (2011).

[b30] Koshino M. Electronic transmission through AB-BA domain boundary in bilayer graphene. Phys. Rev. B 88, 115409 (2013).

