# Supplementary material for: Direct imaging of topological edge states at a bilayer graphene domain wall
Source: Nat Commun. 2016 Jun 17;7:11760. doi: 10.1038/ncomms11760 (PMC4915022; doi:10.1038/ncomms11760)
Supplement: Supplementary Information — Supplementary Figures 1-11 [file ncomms11760-s1.pdf]

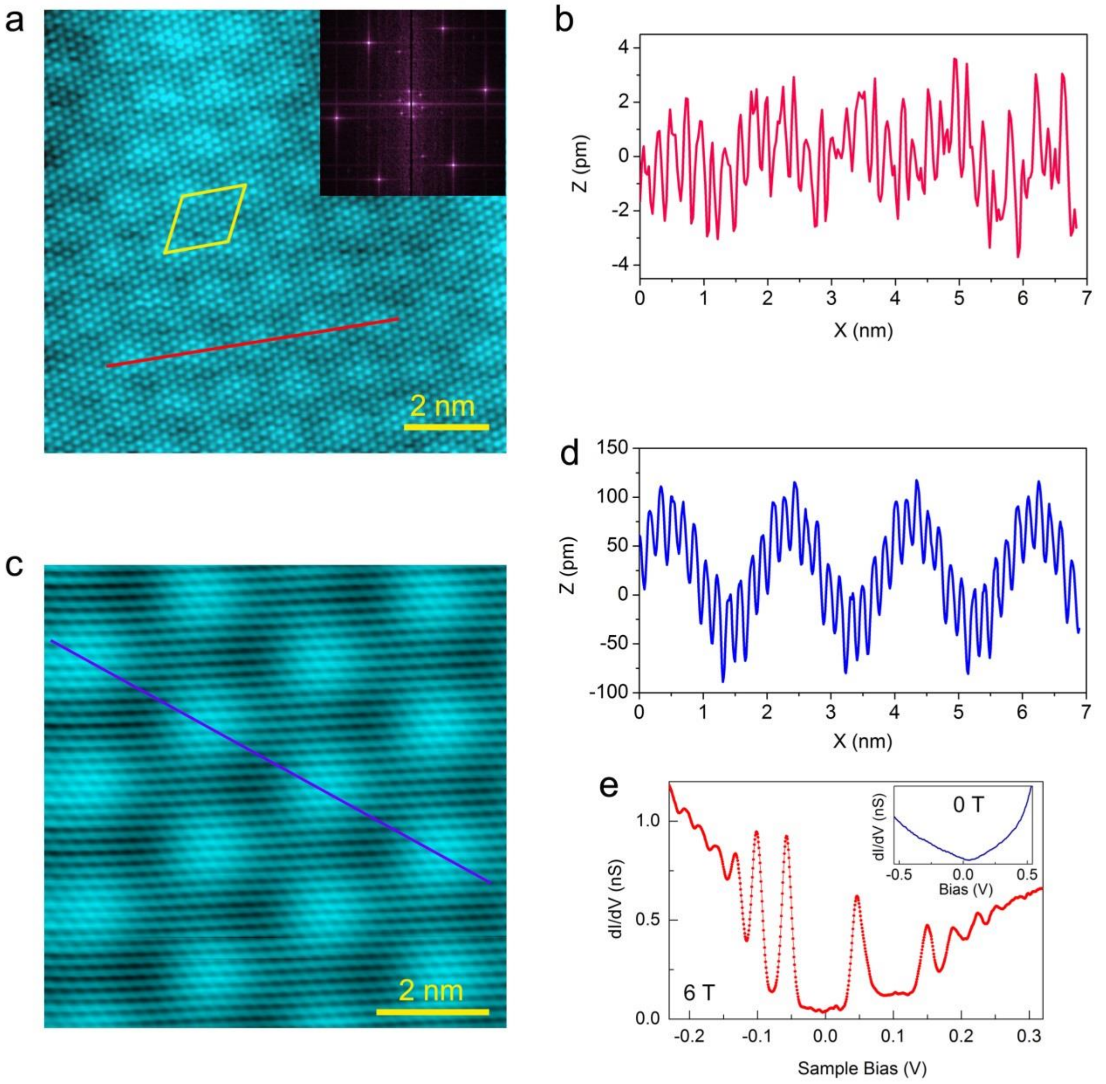

Supplementary Figure 1. Decoupled graphene bilayer and monolayer on graphite surface. (**a**) STM topographic image of the flat region in Fig. 1c, which shows moiré patterns structures with the period of 1.4 ± 0.1 nm (the twisted angle between the topmost bilayer and the substrate is estimated as 10.0 ± 0.5°). The large twisted angle between the topmost bilayer and the substrate results in the electronic decoupling of the topmost bilayer sheet. The triangular contrasting of the STM image demonstrates that the topmost bilayer graphene is Bernal-stacked. Inset: 2D Fourier transforms of the STM image. The out and inner six spots correspond to the reciprocal lattices of graphene and the moiré patterns, respectively. (**b**) Height profile along the red line indicated in (a) exhibits very small corrugation, which indicates that the moiré structures do not arise from the rotation between the topmost bilayers. (**c**) Decoupled graphene monolayer resulted from a twisted angle ~7° between the top single-layer and the substrate. The atomic resolution STM image shows hexangular lattices. (**d**) The height corrugation of the moiré patterns is relatively large, ~0.2 nm. (**e**) A representative spectrum recorded at 6 T in (c) shows Landau quantization of massless Dirac fermions, as expected to be observed in graphene monolayer.

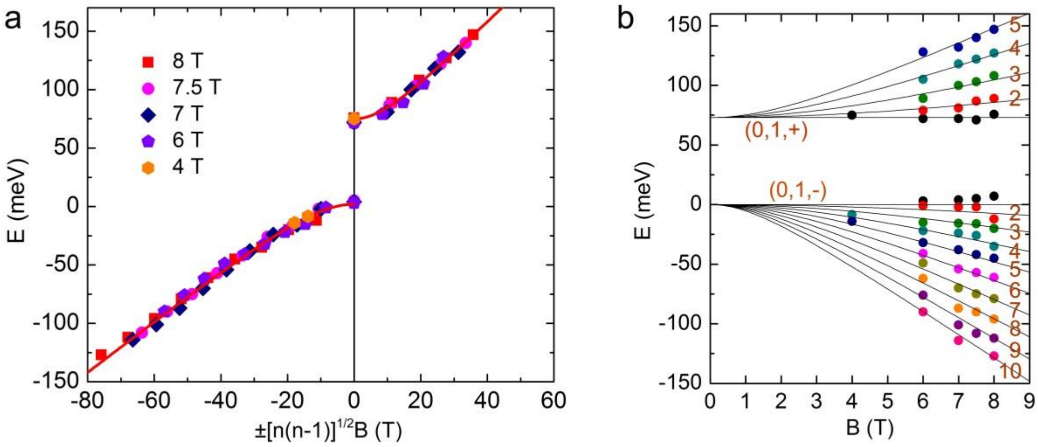

Supplementary Figure 2. Landau quantization of the BA-stacked domain (Fig. 2b). LLs peaks energies extracted from Fig. 2b plotted versus $\pm[n(n\text{-}1)]^{1/2}$ B (**a**) and the magnetic fields B (**b**). The curves are the fitting to Eq. (1). In the Eq. (1), there are three parameters unknown, i.e. E_C, m^* (effective mass of quasi-particles) and U (interlayer bias). Firstly, we can obtain the value of E_C through measuring the two lower LLs peaks. Then, we fitted the data to Eq. (1) with two fitting parameters m^* and U and obtained $m^* = (0.045 \pm 0.001)m_e$ and $|U| = 77 \pm 3$ meV. The value of $|U| \approx E_g$, agrees well with that measured from the energy difference of $LL_{(0,1+)}$ and $LL_{(0,1,-)}$ in the spectra. Similar analysis is used in Fig. 2a for the AB-stacked domain and the corresponding results are presented in Fig. 2d and 2e.

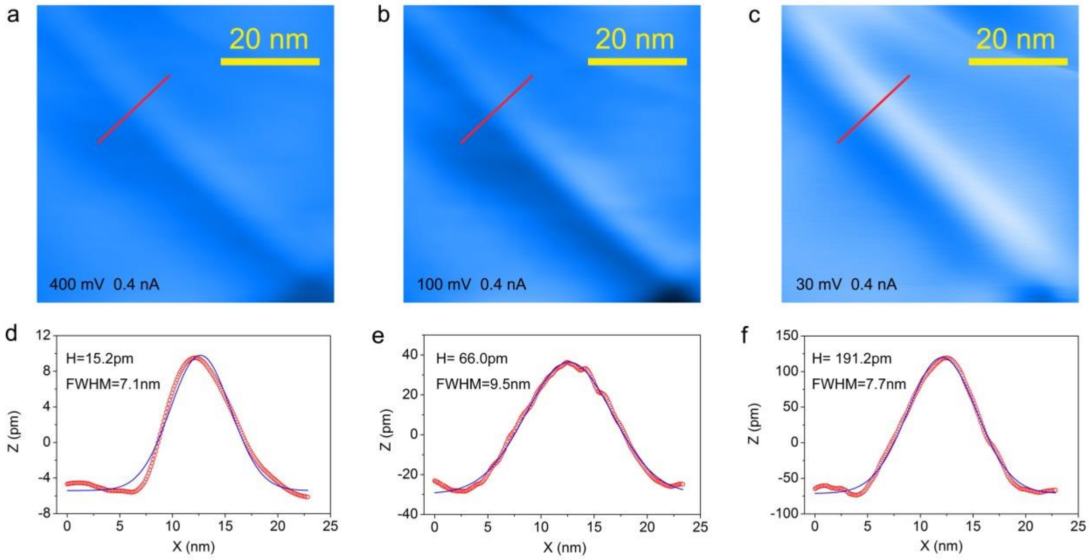

Supplementary Figure 3. Bias dependent corrugation of the AB-BA domain wall. (**a-c**) STM topographic images of the AB-BA domain wall measured with different sample bias 400 mV (a), 100 mV (b) and 30 mV (c). (**d-f**) show height profiles across the red lines in the same position indicated in (a-c), respectively. The red curves are the measured data and the blue curves are Gaussian Fittings. From measuring the height profiles at different positions of the domain wall in (a-c), we obtained the average height about ~ 20 pm (a), ~ 75 pm (b), ~ 210 pm (c) for the domain wall under different bias. The measured height varies dramatically, indicating that the corrugation of the domain wall measured in the STM image is mainly contributed from the LDOS. The average width of the AB-BA domain wall is estimated to be about 8 $\pm$1 nm.

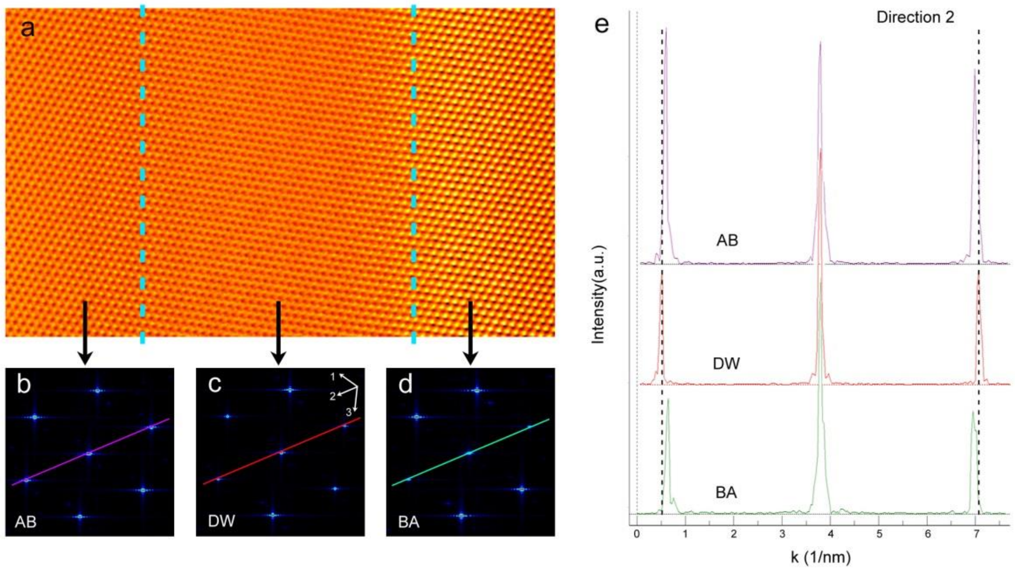

Supplementary Figure 4. The interatomic distance analysis by 2D Fourier transforms of the AB-BA domain wall. (a) Atomic resolution STM current image of the AB-BA domain wall. The dashed lines mark the edges of the domain wall. (**b-d**) Corresponding 2D Fourier transforms of the atomic resolution image in the AB region, the domain wall, and the BA region, respectively. The six bright spots are reciprocal lattices of graphene. Inset of c shows three directions in which the graphene interatomic distances are measured in Fourier images. (**e**) Section views in direction-2 along the colored line indicated in (b-d). The dashed lines label the peaks obtained in the domain wall region. The spacing of the Fourier peaks in direction-2 of the domain wall region is slightly larger than that of the AB and BA regions. The difference is measure to be ~1.5 ±0.5% between them.

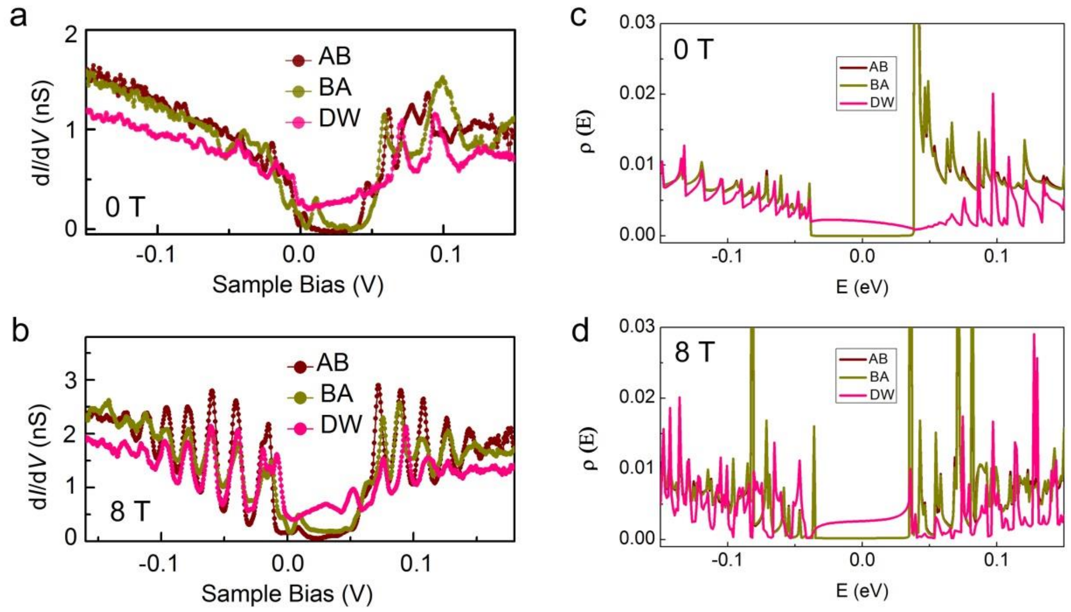

Supplementary Figure 5. Comparison of the DOS spectra in three diffenent regions of the domain wall. (**a** and **b**) Experimental STS spectra measured in three different regions at 0 T and 8 T. (**c** and **d**) Calculated DOS spectra for AB, BA and DW regions under 0 T and 8 T. The calculated LLs under 8 T in domain wall nearly imitate the sequences of that in AB and BA regions, which is agree well with the experimental results. Because of computational limitations, the calculated systems are too small to avoid the emergence of van Hove singularities, as shown in c and d panels, due to quantum confinement.

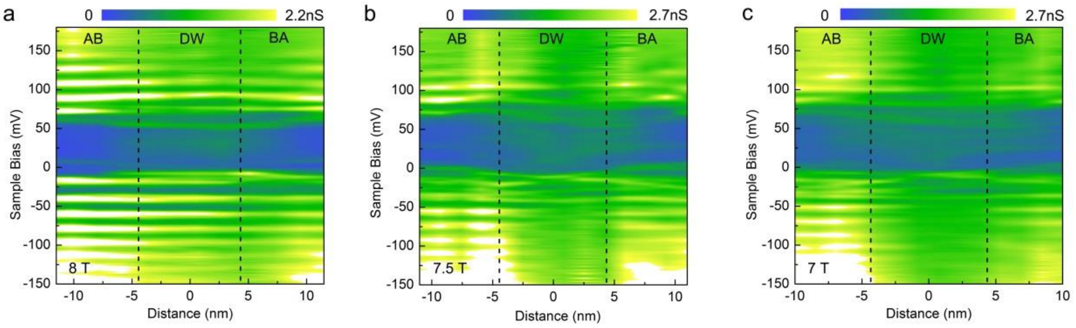

Supplementary Figure 6. Spatial resolved tunneling spectra maps across the domain wall. The spatial resolved *dI/dV* spectra maps are measured at 8 T (**a**), 7.5 T (**b**) and 7 T (**c**) across the AB-BA domain wall. The LLs shown in the domain wall region almost follow the sequences of that recorded in the gaped AB/BA bilayer region. The dashed lines indicate the edges of the domain wall.

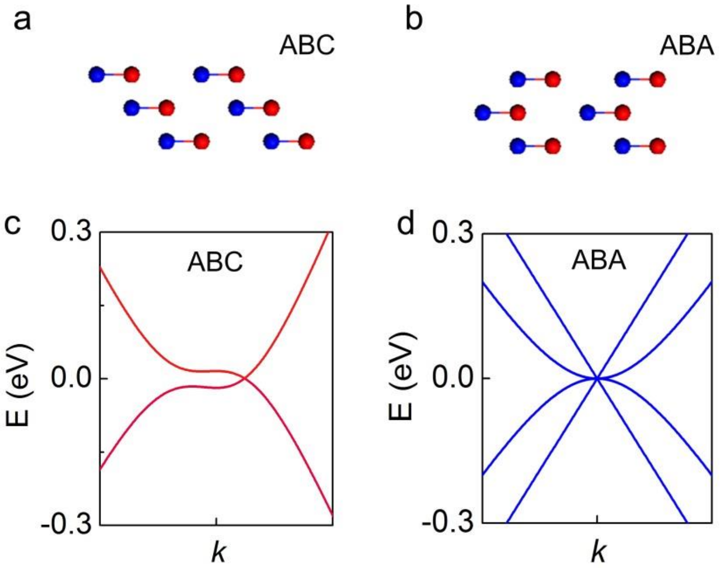

Supplementary Figure 7. Band structures of ABC and ABA graphene trilayers. (**a** and **b**) Schematic structures of the ABC and ABA graphene trilayers. (**c** and **d**) Low-energy band structures of ABC and ABA trilayers. The flat bands appear near zero-energy in ABC trilayer band. The band structure of ABA trilayer is composed of two linear and two parabolic dispersion bands in low energy.

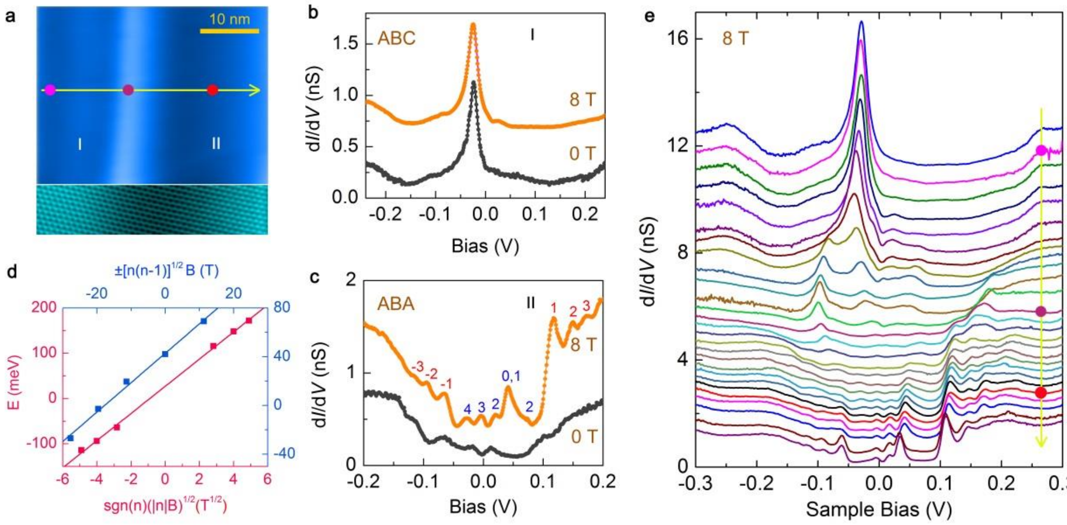

Supplementary Figure 8. ABC-ABA domain wall in graphene trilayer on graphite surface. (**a**) Upper: A STM topographic image of a trilayer graphene with an ABC-ABA domain wall (bright region) on graphite surface (V_b = 0.2 V, I = 0.2 nA). Lower: atomic resolution STM image of the ABC-ABA trilayer domain wall. A transition from triangular lattice (in the ABA region) to hexangular-like lattice (in the center of the domain wall) and then to triangular lattice (in the ABC region) is clearly observed. (**b** and **c**) Typical STS spectra taken at left (b) and right (c) regions of the bright domain wall in (a) under different magnetic fields. The pronounced tunneling peak near zero-bias shown in (b) is generated by the low-energy flat band of the ABC

trilayer graphene, as reported previously. Two sets of LLs for massless and massive Dirac fermions are observed in the ABA trilayer graphene, as marked by red and blue indices. (**d**) LLs peak energies of the massless and massive Dirac fermions show the linear dependence against $\mathrm{sgn}(n)(|n|B)^{1/2}$ (red data) and $\pm[n(n-1)]^{1/2}\,B$ (blue data) respectively. This is consistent with that expected to be observed in the ABA trilayer graphene. (**e**) Spatial variation of the tunneling spectra recorded at 8 T along the line in (a).

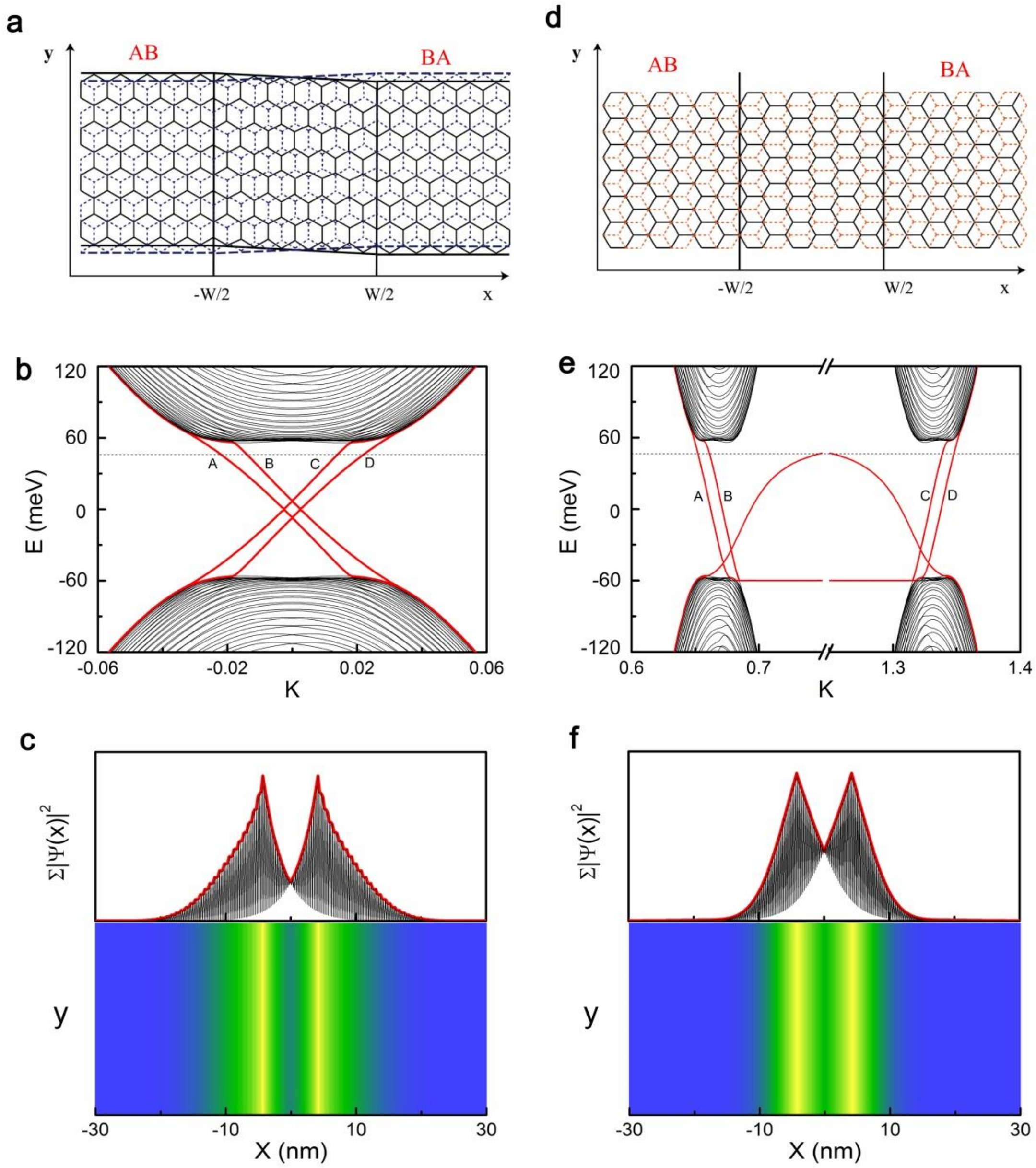

Supplementary Figure 9. Topological chiral states and their distribution at the AB-BA domain walls. (**a**) Illustration of an AB-BA domain wall with armchair edges. (**b** and **c**) Theoretical band structure and spatial distribution of the topological states around the domain wall in (a). (**d**) Illustration of an AB-BA domain wall with zigzag edges. (**e** and **f**) Theoretical band structure and spatial distribution of the topological states around the domain wall in (d).

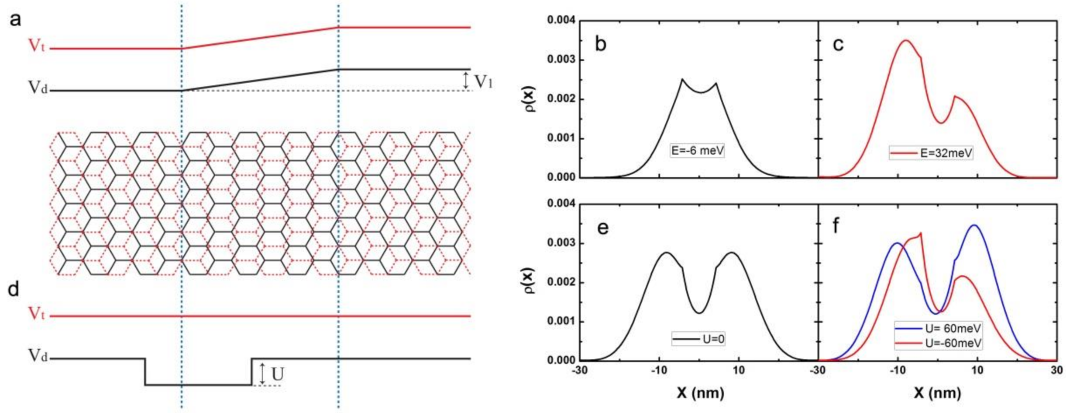

Supplementary Figure 10. Asymmetrical spatial distribution of the edge states. (**a-c**) CNP shifting induced different distribution of the edge states under E = -6 meV (**b**) and E = 32 meV (**c**) with the shift potential V_1 = 10 meV at zigzag AB-BA domain wall. It shows that the symmetry of the edge states in x direction rely on the energy distinctly. (**d-f**) Disorder induced variation of the edge states distribution with different disorder potential U. Obviously, the spatial distribution of the edge states become asymmetrical under finite potential U (**f**) (E = 32 meV both for **e** and **f**).

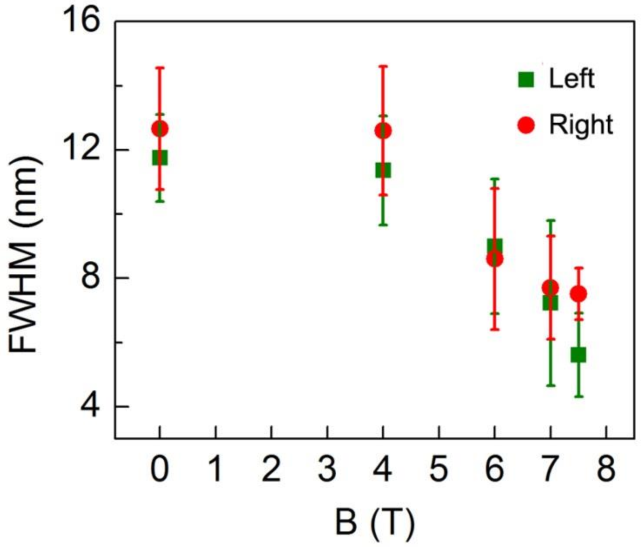

Supplementary Figure 11. FWHM of topological states under various B. The FWHM (full-width at half-maximum) of topological edge states along the two edges of the domain wall display decreasing with increasing the magnetic fields. The green and red dots correspond to the data taken at left and right edges states of the domain wall respectively.
